# Hidden Treasures

**Published:** 1879-07

**Authors:** W. W. Hall


### HIDDEN TREASURES.

"I HAVE meat to eat that ye know not of," was the declaration of the Blessed Redeemer to his wondering disciples. There was a double meaning in this case—the chief was, that he had sources of happiness of which they were ignorant; those divine contemplations, as to the objects and results of his mission, which at the time they could not appreciate, any more than at this day can irreligious men conceive of the joys which belong to the children of the kingdom; the joys of contemplating sin forgiven, of soul saved. On topics like these, a Christian may muse and feed by the hour; and, like Moses in the mount, may come out from that communion with face and soul all bright with a radiance divine.

But these are not the "Hidden Treasures" of the heading. It is intended to speak of treasures worth more than gold to their possessors, they being ignorant of them, while others are enviously aware of their existence.

"I wish I were a Christian; they are the happiest people in the world. I would give all I am worth to be one." Such was the frank declaration of one of the "men of the time," a "representative" man, in a casual conversation on the street, within a few days.

He was a scholar, a genius, a man of renown; and yet few men are more profane, scoff more, vituperate more, when speaking or writing in reference to the men and ministers of our holy religion. He explained himself to mean, that religious people were happy in believing, without a doubt, as to the truth of religion; in regarding the Bible as the book of God, with that implicit confidence which questions nothing; and in their patient looking beyond to "the rest prepared for the" faithful.

To be able to read the Bible, and receive every assertion as of divine origin; to have no misgiving as to the truth of a single word; and even when an incomprehensible declaration is made, to be able to pass on to others more plain, with the calm and quiet feeling that it admits of a perfectly clear and satisfactory explanation; that, as to every declaration of grace, as well as act of Providence,

"God is his own interpreter,
And He will make it plain;
Tho' unbelief is sure to err,
And scan His work in vain"—

to have such a faith abide in the heart from infancy to age, through all the trials and the toils of time, who shall say that it is not "a well of water, springing up unto everlasting life?"

There are some—few, we hope—who, in taking up the Bible, seem to be on the look-out for objections. Instead of reading it with the ready acquiescence in its statements, which they yield, without an effort, to a newspaper, magazine, or new book, only halting at declarations which challenge incredulity, or which are strikingly contrary to personal conviction or experience, they proceed as if they were expecting some absurdity, or some untruth, or some lurking trap or snare. They put upon the Divine declarations their own hasty and unfledged interpretations, and then exclaim, "How can that be?" Such a spirit, in reading the Bible, entitles its possessor to our sincerest commiseration and pity. The whole life long of such a man must be a cloud, whose end is to settle in the darkness of the second death!

A child-like implicitness, in receiving the statements found in the Sacred Scriptures, must be built up at the time when children, as yet, have that same kind of reliance in the truth of all that their parents say. To the good and happy child of a consistent parent, there never is a thought, a feeling, or desire, to go beyond a simple "Father said so." "Mother told me that." To kindle and cherish in a child's heart such a feeling, in reference to the Bible declarations, ought to be the early effort of every parent; and to succeed in doing it, will contribute more to that child's happiness in this life, to say nothing of that which is to come, than the bequest of a fortune. But as to most parents, how many years of self-denying toil are spent in securing such a fortune! how few hours, in that training, which, as to the Bible, leads to "have faith in God!"

Let children and others be taught some first principles as to the Bible.

*First.* The Bible was not written to communicate scientific truths; it only makes use of them as illustrations, or as vehicles of information in reference to God; but, in doing so, it never makes a mistake, by taking for a truth, in science or history, what was not a truth.

*Second.* Although statements may be made which cannot be reconciled with present knowledge, or which are apparently contradictory, a close investigation will always show a beautiful harmony; or additional information will always substantiate the Bible record, or afford ground for rational and satisfactory explanation.

By searching out instances of this sort, from time to time, a growing confidence will spring up in the minds of the young, which will render good service in after life. For example:

Ezekiel, chapter twelve, declares that the king, Zedekiah, of Jerusalem, should die in Babylon—a most unlikely thing! but more, while Zedekiah was not in Babylon, and while he was not to go there—still he was to die there, and yet was not to see Babylon. The quibbler would say, How was he to get to Babylon from Jerusalem without going there? And how could he be in Babylon, and die there, and not see it? The prophet's words are these: "I will bring him to Babylon, yet shall he not see it, though he shall die there." Four years later, a few words in Second Kings, chapter twenty-five, make the whole as plain as day, and of a literal truthfulness that delights the Christian and amazes the infidel; for the king of Babylon came near to Jerusalem, and by his army took Zedekiah captive; took him by force towards Babylon; but before they reached the city, they "put out the eyes of Zedekiah, bound him with fetters of brass, and carried him into Babylon," where he ultimately died.

Volumes could be written, giving such exemplifications as this of the beautiful truthfulness and critical exactness of scripture histories

W. W. HALL,

